# Supplementary material for: Mobile phones and infant health at birth
Source: PLoS One. 2023 Sep 14;18(9):e0288089. doi: 10.1371/journal.pone.0288089 (PMC10501678; doi:10.1371/journal.pone.0288089)
Supplement: S1 File — (DOCX) [file pone.0288089.s001.docx]

**SUPPORTING INFORMATION**

**S1 Table:** Demographic and Health Survey (DHS) waves included in the analysis for each country.

| **Country** | **DHS Wave** |
| --- | --- |
| Albania | 2017-2018 |
| Angola | 2015-2016 |
| Bangladesh | 2017-2018 |
| Benin | 2017-2018 |
| Burundi | 2016-2017 |
| Cameroon | 2018-2019 |
| Ethiopia | 2016 |
| Gambia | 2019-2020 |
| Guinea | 2018 |
| Haiti | 2016-2017 |
| Indonesia | 2017 |
| Jordan | 2017-2018 |
| Liberia | 2019-2020 |
| Mali | 2018 |
| Malawi | 2015-2016 |
| Nigeria | 2018 |
| Nepal | 2016 |
| Philippines | 2017 |
| Pakistan | 2017-2018 |
| Rwanda | 2019-2020 |
| Sierra Leone | 2019 |
| Senegal | 2017-2018-2019 |
| Tajikistan | 2017 |
| Timor Leste | 2016 |
| Tanzania | 2015-2016 |
| Uganda | 2016 |
| South Africa | 2016 |
| Zambia | 2018-2019 |
| Zimbabwe | 2015 |

***Notes***: DHS=Demographic and Health Surveys

**S2 Table:** Attrition analyses between women with and without complete birth-weight information.

|  | **Complete BW** | **Missing BW** | **Difference** |
| --- | --- | --- | --- |
| Mobile-phone ownership | 0.6204 | 0.3656 | -0.2548*** |
|  |  |  | (0.0024) |
| Age | 28.40 | 28.30 | -0.0928** |
|  |  |  | (0.0206) |
| Years of education | 7.042 | 3.317 | -3.7251*** |
|  |  |  | (0.0236) |
| Secondary education (or >) | 0.4908 | 0.2103 | -0.2805*** |
|  |  |  | (0.0024) |
| Wealth ("richer" or >) | 0.4381 | 0.2153 | -0.2228*** |
|  |  |  | (0.0024) |
| Urban | 0.4372 | 0.2195 | -0.2177*** |
|  |  |  | (0.0024) |
| N | 108,103 | 62,813 |  |

***Notes***: BW=birth weight. Standard errors in parentheses clustered at the PSU level. DHS sampling weight applied. *** p<0.01, ** p<0.05, * p<0.1.

**S3 Table**: Association between mothers’ ownership of mobile phones and infants’ birth weight and LBW, births over the previous year, full specification.

***Notes***: LBW=low birth weight. Standard errors clustered at the PSU level in parentheses. DHS sampling weight applied. Country, year, and birth-order dummies omitted from the table. *** p<0.01, ** p<0.05, * p<0.1.

**S4 Table**: Association between mothers’ ownership of mobile phones and infants’ birth weight and LBW, estimates limited to the most recent birth, full specification.

***Notes***: LBW=low birth weight. Standard errors clustered at the PSU level in parentheses. DHS sampling weight applied. Country, year, and birth-order dummies omitted from the table. *** p<0.01, ** p<0.05, * p<0.1.

**S5 Table**: Association between mothers’ ownership of mobile phones and infants’ birth weight and LBW, controlling for PSU fixed effects, full specification.

***Notes***: LBW=low birth weight. Standard errors clustered at the PSU level in parentheses. DHS sampling weight applied. Country, year, and birth-order dummies omitted from the table. *** p<0.01, ** p<0.05, * p<0.1.

**S6 Table**: Association (Average Treatment Effect, ATE) between mothers’ ownership of mobile phones and infants’ birth weight and LBW, using matching techniques.

***Notes***: LBW=low birth weight. SE=standard errors. Standard errors clustered at the PSU level in parentheses. Column 1 presents nearest-neighbor (nn) matching (left) with Mahalanobis distance metric, matching on respondent’s education, rural/urban, wealth index, and country. Exact matches were requested by country and rural/urban. On the right are shown coarsened exact matching (cem) coarsening on the same covariates as in nn. Nearest-neighbor matching with distance based on propensity score differences or Mahalanobis distance involves running through the list of treated units and selecting the closest eligible control unit to be paired with each treated unit. It is the most common form of matching used. Coarsened exact matching is a form of stratum matching that involves first coarsening the covariates by creating bins and then performing exact matching on the new coarsened versions of the covariates. DHS sampling weight applied. *** p<0.01, ** p<0.05, * p<0.1.

**S7 Table**: Association between mothers’ ownership of mobile phones and infants’ LBW, testing sensitivity to missing data (bounding), full specification.

***Notes***: LBW=low birth weight. Standard errors clustered at the PSU level in parentheses. DHS sampling weight applied. Country, year, and birth-order dummies omitted from the table. *** p<0.01, ** p<0.05, * p<0.1.

**S8 Table**: Association between mothers’ ownership of mobile phones and infants’ birth weight and LBW, controlling for mother’s weight, full specification.

***Notes***: LBW=low birth weight. Standard errors clustered at the PSU level in parentheses. DHS sampling weight applied. Country, year, and birth-order dummies omitted from the table. *** p<0.01, ** p<0.05, * p<0.1.

**S1 Fig.**: Estimated mobile-phone coefficient by country (mobile-phone ownership*country interaction) for birth weight (top panel) and LBW (bottom panel)

***Notes***: LBW=low birth weight. 90 percent confidence intervals provided.

**S2 Fig.**: Interaction effects between individual-level mobile-phone ownership and country-level proxies of infant health (predicted margins), predicting LBW

***Notes***: LBW=low birth weight. Estimates of LBW obtained through UNICEF. All other estimates obtained from the DHS StatCompiler. The DHS estimates pertain to all women in the sample (not to the analytical sample of this study). UNICEF estimates are from 2015. DHS estimates are from the same year as the DHS survey. Overlapping bands mean that estimates are not statistically different between the group of women with and without mobile phones.
