# Supplementary material for: Mobile phones and infant health at birth
Source: PLoS One. 2023 Sep 14;18(9):e0288089. doi: 10.1371/journal.pone.0288089 (PMC10501678; doi:10.1371/journal.pone.0288089)
Supplement: Attachment — Submitted filename: Response to Reviewers.docx [file pone.0288089.s002.docx]

**Answer to the Reviewers’ reports on Manuscript ID PONE-D-22-35074 – “Digital Technologies and Children’s Health at Birth,” now retitled “Mobile Phones and Infant Health at Birth.”**

Dear Associate Editor and Reviewers,

We are very grateful for the timely and careful reviews and very useful comments received both by the Associate Editor and the two Referees. We were also pleased to see that many elements of the manuscript were highly appreciated.

In revising the paper, we have taken all suggestions under consideration, and we believe the paper has greatly benefitted from all comments, suggestions, and criticisms. We hope to have adequately responded to the comments received and we are very much looking forward to receiving your valuable feedback on this revised version of the manuscript, which we hope may proceed to the next stage in the publication process. Alongside the Reviewers’ comments, we have also introduced some additional robustness checks/ancillary analyses in light of further feedback received during the 2023 Population Association of America (PAA) annual meeting, which took place in April 2023 (at the time of receiving this revision).

In what follows, we will offer a point-by-point answer to each comment provided by each of the two Reviewers. Before doing that, though, we briefly summarize the major changes introduced in this revision using bullet points:

- Changed the title to better reflect the topic/content of the paper;
- Significantly revised the abstract to reflect new analyses;
- Added some additional relevant pieces of literature;
- Strengthened the theoretical background in three directions: 1) the focus on birth weight as outcome; 2) the focus on intergenerational dynamics; 3) the “Mechanisms” part;
- Redid all figures to make them clearer and more understandable;
- Added multiple new analyses and robustness checks including:
  - Analyses on the sample including last birth only (instead of all births over past 3 years)
  - Analyses on the sample of all births occurring on the previous year
  - Analyses including a wealth index in continuous form
  - Analyses controlling for mother’s weight in kg
  - All analyses now including sex of the child as additional control
  - New analyses revisiting the mechanisms
  - Analyses of “bounds” to make sure attrition is not driving the estimates
  - Robustness checks with fixed-effects for primary sampling units (PSU)
  - Robustness checks using nearest-neighbor matching and coarsened exact matching
  - Heterogeneity analyses at the individual level by level of education and rural/urban
  - Heterogeneity analyses at the country-level to corroborate the idea that results are strongest where infant health is poorest (using different measures of infant health at the country level) and where mobile-phone diffusion is higher
- Added a table of descriptive statistics of women in the manuscript and another table detailing the DHS survey waves used in the Appendix
- Attached a clean code that reproduces our analyses

We also attach a fully revised manuscript in track changes, as well as a clean manuscript with all track changes accepted.

**Editor:**

*Two reviewers have revised the manuscript noting that there are problems that need to be raised. One particular concern is the distance between the title and the actual analysis. Consider changing the title to something more concrete, since the paper is not so much about "digital technologies." Also note the need to provide a better reasoning of the underlying theory.*

Response 1: Thank you very much for your constructive feedback. We have now revised the title to reflect that we are just focusing on cell phones and not on digital technologies more broadly. The new title is as follows: “Mobile Phones and Infant Health at Birth.” We have also strengthened the underlying theoretical mechanisms we have in mind, as well as the motivation for 1) focusing on an intergenerational perspective and 2) choosing birth weight as an outcome. All in all, we have made very substantive revisions both to the text and to the analyses, which are now much more robust.

**Reviewer #1:** 

*Thank you for the opportunity to review the manuscript. It is incredibly insightful, analyses are very well done and easy for any reader to follow. Bravo! A few comments for consideration*

Response 1: We very much thank the Reviewer for such enthusiastic comments and for providing additional valuable feedback, discussed below.

………………………………………………………………………………………………………………...

*Abstract:
1) please elaborate on what " shape global development outcomes" means...could be more specific in stating that it shapes the SDG outcomes and reference the WHO's digital health agenda to strengthen the statement
2) POWERFUL! "Partly, this association
is mediated by a higher number of antenatal visits and by the extent to which mothers
hear about family planning by text message."
3) Also VERY powerful! "Results are stronger in countries where
infant health is poorest, suggesting the comparatively higher potential of the diffusion of
digital technologies for global development in poorest contexts."*

Response 2: Thank you for all these suggestions. The abstract has been revised accordingly taking all suggestions into consideration. We have now mentioned the SDGs in the abstract and specified that when we talk about digital technologies in this work we are referring to mobile phones. We have also clarified that this work only identifies associations.

………………………………………………………………………………………………………………...

*Intro:
1) See comment #1 of abstract critique. Done BEAUTIFULLY in abstract
2) The field is slowly moving away from the term “sub Saharan Africa” due to its negative connotation and instead using “Africa south of the Sahara” or “The African region” or if need be, “resource-limited settings of the African region “ as not all of the region is as “poor” as is often depicted.
Please see resources below:
"The term spread as a replacement for the racially-tinged phrases “Tropical Africa” and “Black Africa” that were used until around the 1950s, says Columbia University anthropologist Brian Larkin."*
[*http://www.cnn.com/2010/WORLD/africa/09/20/sub.saharan.africa/index.html*](https://urldefense.proofpoint.com/v2/url?u=http-3A__www.cnn.com_2010_WORLD_africa_09_20_sub.saharan.africa_index.html&d=DwQGaQ&c=slrrB7dE8n7gBJbeO0g-IQ&r=tlnbREEYduQAM97n1HGfkKK3ljTJvehygMs87RIKTT0&m=8jgVrhAfBdNNd2AVIgQyQC-K-s8xSw3yNPJ9UeMUMmBuVKF4fofQiC0rilGhLadd&s=wx1z0YC2eXnjwzTRtBLKs8Il4oEEDCdeDwsQ5ZkYFBQ&e=)
[*https://qz.com/africa/770350/why-do-we-still-say-subsaharan-africa*](https://urldefense.proofpoint.com/v2/url?u=https-3A__qz.com_africa_770350_why-2Ddo-2Dwe-2Dstill-2Dsay-2Dsubsaharan-2Dafrica&d=DwQGaQ&c=slrrB7dE8n7gBJbeO0g-IQ&r=tlnbREEYduQAM97n1HGfkKK3ljTJvehygMs87RIKTT0&m=8jgVrhAfBdNNd2AVIgQyQC-K-s8xSw3yNPJ9UeMUMmBuVKF4fofQiC0rilGhLadd&s=iFjYcLf2zKIO47dtoNQV1TXkLWcQ5kSOkMdBQHFhilo&e=)

Response 3: Thank you. We have now explicitly mentioned the WHO digital health agenda in the introduction and we have removed the term sub-Saharan Africa, in line with the resources provided by the Reviewer.

………………………………………………………………………………………………………………...

*"little research to date has focused on the intergenerational implications of digital technologies for child health and wellbeing" why is an intergenerational focus important? please elaborate a little more*

Response 4: We have now further elaborated on why an intergenerational perspective may provide valuable insights.

………………………………………………………………………………………………………………...

*Similarly, why is the infant's birth weight (of all the metrics available for infant health), important as a relevant outcome for the field? What does birth weight predict for child development and other relevant outcomes?
"– bears any relationship with their offspring
health at birth as measured, for instance, by birth weight."*

*and this: "no study has focused explicitly on the relationship between mothers’ mobile-phone ownership and infants’ health as measured by birth weight and low birth weight (LBW) as we do in this study."*

Response 5: We thank the Reviewer for this important comment. Birth weight has been shown to be one of the most relevant and precise predictors of later-life health and education across multiple contexts. We have added some motivation and cited relevant papers to support our decision – this is now featured in the revised introduction. We have also removed the vague phrasing “for instance” which sounded like we picked this outcome randomly.

………………………………………………………………………………………………………………...

*Beautiful section "EXISTING EVIDENCE FROM LMICs ON THE ROLE OF MOBILE PHONES IN
THE HEALTH ARENA"*

Response 6: Thanks! We have made some edits to this section to incorporate some comments and suggestions made by Reviewer 2.

………………………………………………………………………………………………………………...

*I'd remove the "now" in this sentence "mothers can now call a helpline to inquire and ask questions about their child’s health conditions"*

Response 7: Done.

………………………………………………………………………………………………………………...

*Not sure why the "so called" is necessary in these phrases? "highlighting again access (so-called, first-level) and skill-related (so-called, second-level) digital divides" Also those need references.*

Response 8: Edited and reference added.

………………………………………………………………………………………………………………...

*Methods
Why were Armenia and Maldives excluded from the final sample selection? What was the reason?*

Response 9: This is stated in the manuscript in the above sentence. We apologize if we had not been clear enough. The reason has to do with the fact that in those two countries, over 95% of women own mobile phones, hence there would not be enough variability in our main predictor of interest. We excluded all countries with close-to-complete mobile-phone penetration.

………………………………………………………………………………………………………………...

*Beautiful discussion of reverse causality
Great discussion of sampling bias with the "advantaged" mothers*

Response 10: We are glad the Reviewer appreciated these discussions. We have now also strengthened the analyses of attrition by testing the sensitivity of the estimates to assuming that all “missing births” are low birth weight (LBW) – in line with a valuable suggestion provided by Reviewer 2 (**Table A7**). Results are robust.

………………………………………………………………………………………………………………...

*The authors have no knowledge of whether this phrase is true or not, so I'd remove
" (twins, most likely)."*

Response 11: Dropped.

………………………………………………………………………………………………………………...

*The methods are nice and clear and easy to follow. for example this is BEAUTIFUL
"In other words, over the previous three years, 170,916 mothers had 260,158 births,
of which only 152,172 have complete birth-weight information"*

Response 12: Thanks for the enthusiastic comments.

………………………………………………………………………………………………………………...

*This phrase does not make sense to the Reviewer. How does 2% difference range translate to 6% and 25% difference? Also, 2% is not a statistically significant difference...whether raw or adjusted...perhaps the solution is to translate this 2% difference to population level data. 2% represented XYZ number of babies in Bangladesh and XYZ number of babies Albania...is that what the authors are trying to say? Would make more sense to the reader if so.*

*As the variability in LBW is high across countries, a 2 percentage-point difference may imply percentage changes ranging between 6% in Bangladesh to 25% in Albania."*

Response 13: We have clarified this point. We are talking here about 2 percentage points, not 2%. 2 percentage points may or may not be a significant difference (in fact, in our analyses it is, but this is not shown yet at this stage). In this case, we are simply presenting raw descriptive statistics (we are not adopting a regression framework yet). Any percentage-point difference can be translated in % terms by referring to the benchmark (i.e., the mean LBW among individuals with no mobile phones, which is roughly 17% on the pooled sample). This is how the % difference is obtained from the percentage-point ones. We have now clarified the sentence to provide % differences both for the whole sample and for the range of countries (with a minimum and a maximum). We hope this is now clearer.

………………………………………………………………………………………………………………...  *Table 1: define LBW cutoffs in tables for the reader*

Response 14: Defined in the notes to the Table. Thanks for pointing this out. We have also spelled out all acronyms in the Tables and Figures.

………………………………………………………………………………………………………………...

*Please speak to this a little more by walking the reader through the data for either country as it's a mind-twist:
"What is even more important is that the decline in the proportion of LBW
infants born to mothers with mobile phones is most marked in countries that have some of the
highest probability of LBW infants born to mothers without mobile phones – with two clear
examples being Pakistan and Bangladesh. "*

Response 15: We apologize for this confusion, and we acknowledge that this statement was confusing and not properly supported by adequate evidence, mostly because previous Figure 4 was confusing. The idea is that in some of the countries with highest prevalence of LBW, such as Pakistan, we observe some of the biggest differences between women with and without mobile phones. We have now made examples using both Pakistan and Nepal and walking the reader by providing again the share of LBW births and the estimated coefficients. Estimates are among the most negative in these two countries, which have among the highest share of LBW births. We have also made additional graphs showing this visually in two other ways against country-level measures of infant health, namely LBW at the country level obtained from UNICEF, prevalence of stunting, wasting, and underweight (from DHS). All our results (**Figure 5**), including interaction effects reported in the Appendix (**Figure A2**), support the idea that associations are more negative (hence, results are stronger) in contexts where infant health is poorer (i.e., where LBW is higher, where stunting is higher, etc.). We hope this may be clearer now.

………………………………………………………………………………………………………………...  *Discussion
This phrase needs a reference "corroborating the idea that mobile phones enable smoother communication and better community outreach, which in turn allow women to more swiftly connect with other women in their communities, as well as with health professionals/practitioners/facilities, leading to positive infant health outcomes."*

Response 16: Thank you, references added.

………………………………………………………………………………………………………………...

*The discussion could benefit from some discussion on the importance of the results with regards to child/human development, SDGs, maternal health, access, etc etc etc. Just re-iterating the results is not sufficient. Please see:

1) Anto-Ocrah, M., Latulipe, R.J., Mark, T.E. et al. Exploring association of mobile phone access with positive health outcomes and behaviors amongst post-partum mothers in rural Malawi. BMC Pregnancy Childbirth 22, 485 (2022).* [*https://doi.org/10.1186/s12884-022-04782-0*](https://urldefense.proofpoint.com/v2/url?u=https-3A__doi.org_10.1186_s12884-2D022-2D04782-2D0&d=DwQGaQ&c=slrrB7dE8n7gBJbeO0g-IQ&r=tlnbREEYduQAM97n1HGfkKK3ljTJvehygMs87RIKTT0&m=8jgVrhAfBdNNd2AVIgQyQC-K-s8xSw3yNPJ9UeMUMmBuVKF4fofQiC0rilGhLadd&s=ztfTQ9fCLeiFtLZo6miphBYRLZEYz1jW_Xo3LFjWBHw&e=) *2) Healthy People.gov. Social Determinants of Health. 2014 2019 3/20/2019]; Available from:* [*https://www.healthypeople.gov/2020/topics-objectives/topic/social-determinants-of-health*](https://urldefense.proofpoint.com/v2/url?u=https-3A__www.healthypeople.gov_2020_topics-2Dobjectives_topic_social-2Ddeterminants-2Dof-2Dhealth&d=DwQGaQ&c=slrrB7dE8n7gBJbeO0g-IQ&r=tlnbREEYduQAM97n1HGfkKK3ljTJvehygMs87RIKTT0&m=8jgVrhAfBdNNd2AVIgQyQC-K-s8xSw3yNPJ9UeMUMmBuVKF4fofQiC0rilGhLadd&s=8gcuk6XoMG_eu0tNicDdMC3kPkFoIY2V-O12aunwPRY&e=)*.

3)Petkovic J, Duench S, Trawin J, Dewidar O, Pardo Pardo J, Simeon R, et al. Behavioural interventions delivered through interactive social media for health behaviour change, health outcomes, and health equity in the adult population. Cochrane Database Syst Rev. 2021;5:Cd012932.*

Response 17: Thank you very much for these valuable suggestions, which we have now incorporated and featured more prominently in the discussion section, which we have also strengthened considerably. In saying so, we have nonetheless tried to keep careful in not claiming causality as our design does not allow to do so. Hence, the policy implications are somewhat “limited.”

………………………………………………………………………………………………………………...  *The figures are great but pixelated*

Response 18: We have now provided better-quality figures. Apologies, we provided them in the wrong format before. Relatedly, we have also slightly modified some of the previous figures to make them clearer and more intelligible (e.g., removing duplicate information). A couple figures are completely new (namely, **Figures 4, 5, and 6**), and provide information on the heterogeneity of the estimates at both the individual- and the country-level.

………………………………………………………………………………………………………………...

**Reviewer #2:**

*This study examines the cross-sectional relationship between mobile phone ownership and birthweight. It is an interesting topic; however, I have several comments about the framework/theory of change, methods, and presentation of results.*

Response 1: We very much thank this Reviewer for her/his valuable comments and suggestions. We are grateful for the very deep engagement with our work and thankful for all the suggestions received, which we discuss in sequence below.

………………………………………………………………………………………………………………...

*On page 6, the authors write “Despite the obvious connection between most of these interventions and children’s health outcomes, alongside the myriad opportunities enabled by mobile phones in the health arena, no study has focused explicitly on the relationship between mothers’ mobile-phone ownership and infants’ health as measured by birth weight”. Unfortunately, I do not find this particularly obvious. (Moreover, this sentence comes after a whole paragraph on barriers to using mHealth so it’s not clear what “these interventions” even refers to.) In general, the introduction would benefit from a stronger link between what is known about mobile phones and maternal/reproductive health and how that would theoretically impact birthweight. The authors list a variety of studies in very different areas – HIV, measles, contraception, etc. but it’s not clear how any of these mobile phone programs would impact birth weight. I suggest the authors focus on the relevant studies and country mHealth programs (e.g., measles vaccination is irrelevant as the child would already be born), as well as provide a framework or model to indicate the mechanisms through which those interventions could change birthweight.*

Response 2: The aforementioned sentence has been dropped and rephrased. We have added motivation to explain why we focus on birth weight and why mobile phones may matter for birth-related outcomes, both in the intro and in the background section. We have also engaged more deeply with a discussion of potential mediators/mechanisms. Unfortunately, there is no literature yet on the relationship between mobile phones and birth weight, hence this paper. The claim we are making is that mobile phones have been shown to be instrumental for women’s antenatal care, improved knowledge of health-related issues during pregnancy, etc. hence, given that birth weight is (partly) a reflection of behavior before and during pregnancy, there is reason to expect mobile phones to matter for birth weight through some of the above channels. To better highlight this, we have restructured the section to first describe what is known about mobile-phone diffusion and mobile-phone interventions and health (focusing on a broad definition of health) in LMICs, and then delved into more detail in the relationship between mobile-phone ownership and birth weight (our focus in this study).

………………………………………………………………………………………………………………...

*I agree with the authors that missing birthweight for 36% of the sample is a significant issue. I recommend the use of multiple imputation to impute low birth weight status from the predictor variables. Another alternative is to do a sensitivity analysis in which 100% of those missing are assumed LBW (as a worst-case scenario) and see if results stand up. These observations are clearly not missing at random based on the Table A1, so the analysis should not treat them that way.*

Response 3: We very much thank the Reviewer for this valuable suggestion. Indeed, these observations are not missing at random. These are poorest women, most likely giving birth at home or giving birth in health facilities and/or communities where births do not get registered. As we are not big fans of multiple imputation strategies (which are quite sensitive to minor differences in specifications), we have decided to follow the Reviewer’s second suggestion, i.e., to conduct a “bounding exercise” whereby all missing births are LBW (all missing coded as 1) and all missing births are normal weight (all missing coded as 0). The results from the full models for both extreme-case scenarios are provided in Appendix **Table A7** and Response Table below, **Table R1**. What the results show is that if all the “missing births” were assumed to be normal weight (column 2), the coefficient on mobile-phone ownership would not be predictive anymore (the coefficient would become zero). Note that this is an unlikely scenario as we have shown that these mothers are extremely disadvantaged, hence more likely than average to have LBW births. Conversely, if all “missing births” were assumed to be LBW (column 3), the coefficient on mobile-phone ownership would be three times as strong (-0.051 compared to -0.018) and results would hold even more strongly. Thank you again for this valuable suggestion.

………………………………………………………………………………………………………………...

*The authors include multiple births (siblings or twins) from individual women. This results in clustering in the data at the woman-level. As the exposure is woman-level, I would recommend conducting the analysis that way. For example, the authors could take the most recent birth, or a birth at random for each woman, rather than including all births.*

Response 4: We thank the Reviewer for this additional suggestion. This is precisely the kind of analysis we conducted in the first version of this paper, but we then received multiple suggestions to include all births a woman has had over the past year or over the past three years, which is why we ended up expanding the sample of births and controlling for birth order (as per the current analyses). The rationale is that the aetiology of first versus subsequent births is very different, hence we’d rather keep a mixed sample of births. Ultimately, it does not change much as – as the manuscript states – 87% of women only had one birth over that timeframe. Nonetheless, we had run additional analyses focusing only on the most recent birth and results are virtually identical – even slightly stronger for the most recent birth (now provided in the Appendix **Table A4** and in the response to reviewers here below – **Table R2**). We have also run analyses keeping all births occurring over the previous year (instead of previous three years), and these results can be found below in **Table R3** (as well as in the Appendix **Table A3**). All results are unchanged, hence we decided to keep all births occurring over the past three years, a decision which allows us to retain an adequate sample size, especially for conducting country-specific analyses.

………………………………………………………………………………………………………………...

*The mediator analysis needs more careful attention. First, there is no justification or motivation for the use of these mediator variables (what are the theoretical pathways through which mobile phones could improve birth weight?) This should also be included in the introduction as part of the theoretical framework or model.*

Response 5: We agree with the Reviewer that we had glossed over this section too quickly. We have now added a paragraph where we explain in more detail what the pathways may be (given the available data). We distinguish between: 1) expanded access to reproductive health-care services in the pre-natal (number of antenatal visits) and the post-natal periods (birth assisted by staff); 2) improved access to health-related information (knowledge of place to get HIV tested; knowledge of any contraceptive method, heard family planning by text message); 3) expanded communication and broader social networks (no way to test this with the current data).

………………………………………………………………………………………………………………...

*Second, the first three mediator variables are likely very highly correlated. The number of antenatal visits is not very different from whether the mother saw a health professional for antenatal care. The same goes for visiting a health facility in the previous 12 months, which they would do if they were getting antenatal care. I would not include all three of these into the same model as they are capturing the same thing – access to health care, which is only one pathway. The authors should choose one of these three to capture health care access. The last mediator on hearing family planning text messages is good, though as I mention in point 4 above, it needs to be justified as to what the authors think it is measuring in terms of the mediator pathway. What about other pathways, for example, are there any knowledge variables in the DHS that the authors could explore with regards to family planning/child health? I would imagine that access to a mobile phone could improve knowledge of health. There may be other pathways as well.*

Response 6: Thank you for this comment. So, in general terms it is true that these variables may seem to capture the same construct but, in reality, this is not confirmed by the data. Just look at the following crosstabs of “antenatal visits” and “seeing health professional.” One would expect to see all individuals who had not seen any health professional to fall under zero antenatal visits (or vice-versa), but this is not the case (e.g., because some antenatal visits can be at home).

```
Number of |
antenatal |      Saw health
visits |   professional for
during |     prenatal care
pregnancy |     0. no     1. yes |     Total
-----------+----------------------+----------
0 |    14,157          1 |    14,158
1 |     4,184      1,197 |     5,381
2 |     8,402      2,641 |    11,043
3 |    19,027      5,928 |    24,955
4 |    23,684      5,896 |    29,580
5 |    13,517      3,264 |    16,781
6 |    11,403      2,654 |    14,057
7 |     6,534      1,631 |     8,165
8 |     4,885        972 |     5,857
9 |     4,064        572 |     4,636
10 |     2,017        363 |     2,380
11 |       846         79 |       925
12 |     1,250        165 |     1,415
13 |       386         32 |       418
14 |       356         53 |       409
15 |       395         33 |       428
16 |       209         25 |       234
17 |        68          4 |        72
18 |       195         15 |       210
19 |        41          3 |        44
20 |       375         47 |       422
21 |         7          0 |         7
22 |         7          1 |         8
23 |         2          0 |         2
24 |         7          0 |         7
25 |         2          0 |         2
26 |         1          0 |         1
27 |         5          0 |         5
28 |         2          0 |         2
29 |         1          0 |         1
32 |         5          0 |         5
36 |         1          0 |         1
-----------+----------------------+----------
Total |   116,035     25,576 |   141,611
```

In any case, to make the pathway section more theoretically grounded (and to make it fit into “broad” theoretical categories), we have decided to only keep antenatal visits as a measure of expanded access to reproductive health-care services in the pre-natal period and birth assisted by staff as a measure of expanded access to health-care services in the post-natal period. As for the knowledge variables, there are very few suitable candidates, but we have now included two variables that proxy for women’s knowledge of reproductive health, namely “knows of a place to get tested for HIV” and “know of any modern method of contraception.” These are not related to the birth experience itself, but to health-related knowledge more broadly. All results have been rerun with these new mediators, now further justified in the background section. Also, we have kept as part of this group the receipt of text messages by phone.

………………………………………………………………………………………………………………...

*Results: It would be helpful to have a table of descriptive characteristics of women for the exposure, all controls and all mediator variables.*

Response 7: Thank you. We added a table of descriptive statistics for the analytical sample of women, now **Table 1** in the revised manuscript.

………………………………………………………………………………………………………………...

*There is likely residual confounding by SES, despite controlling for education and wealth index. Perhaps the authors could include an asset index. I see the controls for owns TV and radio, but I believe the DHS includes a list of assets that can be included as an index. That could reduce some of the residual confounding by SES.*

Response 8: Wealth index is precisely what the Reviewer is referring to, which is already included in the analysis. Wealth indices are asset indices in low- and middle-income countries, as there is no information on income/wealth itself. The wealth index comes in two forms: a continuous index built through Principal Component Analysis (PCA) and a categorical variable using wealth quintiles. As the wealth index is built differently for each country, existing research suggests that the categorical variable is more appropriate for a multi-site investigation (see, for instance, Smits and Steendijk 2015) – pretty much in the same way as using education in categories is better than using education in continuous form. As such, we decided to retain the current analyses with the wealth (or asset) index in the present form, yet we provide below results using the wealth index as continuous variable, excluding radio and TV as elements entering the PCA (**Table R4**). As the Reviewer can see, results are virtually unchanged. This said, we agree that there is likely residual confounding by SES. Unfortunately, this is always the case with cross-sectional analyses, and we acknowledged this limitation extensively in the discussion section. Furthermore, to partially address this issue (at least as driven by observable characteristics), we have complemented OLS models with two different matching techniques, namely nearest-neighbor matching and coarsened exact matching (CEM).

………………………………………………………………………………………………………………...

*I would also include mother’s health variables, including weight, self-reported health status, and any other health indicators that are available, as those could be confounders (health and wealth relationship is bidirectional).*

Response 9: Thank you very much for this valuable comment. We do agree with the Reviewer that including these variables would make a lot of sense. However, this is trickier than imagined, for two reasons. First, the DHS are very comprehensive when it comes to health of children at birth and when it comes to women’s reproductive health, yet they are very limited when it comes to measuring women’s general/overall health. For instance, there is no information on women’s self-reported health (this will be included in the upcoming rounds of DHS data collection, namely “DHS-8”). The only measure we could identify was women’s weight (in kg), yet note that this variable is not available in 5 countries, namely Angola, Indonesia, Philippines, Senegal, and Zambia (some of the countries that provide the biggest sample sizes). As such, we have decided to exclude these analyses from the main paper due to significant loss of sample size, yet we have run analyses on 24 countries and included them at the bottom of this letter (please, find them in **Table R5**). These analyses show that mother’s health indeed matters, as the coefficient on mobile-phone ownership is cut in half, yet it remains quite relevant. We have acknowledged this limitation in the conclusions and discussion.

Related to this discussion, while there are lots of measures of women’s reproductive health, we have decided not to include them in the main analyses, as these are very highly correlated with the mediators we include in this study. Hence, we want to keep them away (or separated) from the potential pathways we have in mind.

………………………………………………………………………………………………………………...

*Figure 4 is not interpretable. It’s impossible to see anything in these two figures. The authors could change the margins plot to show a bar plot perhaps, by country. Or else present this information in a table with adjusted prevalences and CIs, which may make more sense than a figure if you are trying to show small differences by country.*

Response 10: Thank you for this suggestion, which also echoes comments from Reviewer 1. We have now dropped this Figure altogether and replaced it with a set of other figures and tables that provide clearer evidence of heterogeneous associations, both at the individual level (by women’s education and household location of residence) and at the country level (using different proxies of infant health). The text has been changed accordingly. Please, see more detailed discussion below.

………………………………………………………………………………………………………………...

*In addition, for Figure 4 I would remove the antenatal visit variable as it is on the causal pathway and, as far as I understand, the authors are trying to estimate the adjusted difference in risk of low birthweight by phone status. Including variables on the causal pathway biases these estimates.*

Response 11: Yes, excellent and very sensible suggestion. All the heterogeneity analyses now – as well as the robustness checks on the analytical sample – are conducted excluding the channels/mediators. Basically, they are run on model 3 in Table 2, which is now our “full specification.” This also allows us to retain a higher sample size.

………………………………………………………………………………………………………………...

*Discussion: The authors wrote “Lastly, we found stronger results in countries where infant health is poorest, suggesting the comparatively higher potential of the diffusion of digital technologies in more disadvantaged contexts.” I don’t see the analysis that shows poorer countries benefit more? Is this just eyeballing it from Figure A1? If that is the case, the authors should conduct this analysis formally, for example, by including country GDP as an interaction variable. This was also repeated in the abstract.*

Response 12: We apologize for the confusion, also expressed in comment 15 by Reviewer 1. We have now dropped Figure 4, which was confusing, and we have replaced it with a series of plots showing interactions between mobile-phone ownership and individual-level proxies of SES such as education and rural/urban (see below) and country-level proxies of SES and health status. For the latter, we have followed two strategies: 1) plotting the resulted estimated association against four different measures of infant health (we chose infant health over GDP per capita as this is a more precise measure in this context – infant health is not always worse in countries with lower GDP per capita) through scatterplots (in the main text, **Figure 5**) and plotting the estimated margins of the interaction terms of the aforementioned country-level variables with individual-level mobile-phone ownership (in the Appendix, **Figure A2**). Our results show that mobile phones matter more where infant health is poorest. Looking at the scatterplots, we observe stronger results (i.e., more negative associations) where country-level LBW is higher, stunting is higher, and underweight is higher. This is confirmed by the predicted margins in the Appendix, which show overlapping confidence intervals in contexts where infant health is good, and emerging differences between owning phones vs not as infant health gets worse and worse.

………………………………………………………………………………………………………………...

*It would also be interesting to examine patterns of country-specific results by other variables. For example, do countries with higher mobile phone ownership have stronger results? Or more urban populations?*

Response 13: We agree with the Reviewer that this would be very interesting, and we are currently undertaking this investigation in another manuscript where we precisely try to explain the heterogeneity in the estimates across countries using different country-level predictors such as Human Development Index, Gender Development Index, Gini coefficient, unemployment, share not in education, etc. As such, we prefer to keep the focus of this manuscript primarily on the micro level. Nonetheless, to partly address the Reviewer’s suggestion, we have added heterogeneity analyses by rural/urban and by mother’s level of education at the individual level, as well as heterogeneity by some country-level measures of health, as detailed above. For the individual-level analyses of heterogeneity, see results below and now also reported in the paper (**Figure 4**). We find strongest coefficients among mothers with no education or primary education, while the coefficient for highly educated women is close to zero (also, wider confidence intervals as there are few individuals with tertiary education). Similarly, we find slightly higher magnitudes in rural areas, yet there are no statistically significant estimates between rural and urban areas. Our findings suggest that mobile phones may be most useful/instrumental/beneficial among the poorest individuals, both at the individual level and the country one. Also, our findings suggest stronger results in countries where mobile-phone ownership is higher, in line with the hypothesis of the Reviewer. We used two measures to test this: mobile-phone ownership at the country level on the whole sample of DHS women (not just mothers with children who have information on birth weight) and mobile-phone subscriptions per 100 people from the World Development Indicators (compiled by the International Telecommunication Union) – this Figure can be found in **Figure 6**.

………………………………………………………………………………………………………………...

*Minor points: The analysis should control for infant sex as there is notable gender difference in birthweight and LBW.*

Response 14: Thank you for this very insightful comment. We have now added a control for the sex of the child, coded as 1 if the child is female and zero otherwise. Indeed, the data provide evidence that girls are more likely to be born LBW, in line with the relevant literature.

………………………………………………………………………………………………………………...

*Please include in the Appendix the year of the DHS survey included for each country so that others may be able to replicate the results.*

Response 15: Thank you for this suggestion, we have now added this Table in the Appendix (**Table A1**), and we have also attached our code for this paper, which we are happy to make publicly available.

**Robustness Tables - Response to Reviewers**

**Table R1**: Robustness checks – bounding exercise for births with missing birth weight

**Table R2**: Robustness checks conducted on most recent birth (regardless on when this birth occurred)

**Table R3**: Robustness checks conducted on all births occurring over the past year, instead of past 3 years

**Table R4**: Robustness checks with wealth index as continuous variable

**Table R5**: Robustness checks including mother’s weight as control (note: five countries get lost)
