# Supplementary material for: Mobile phones and infant health at birth
Source: PLoS One. 2023 Sep 14;18(9):e0288089. doi: 10.1371/journal.pone.0288089 (PMC10501678; doi:10.1371/journal.pone.0288089)
Supplement: Attachment — Submitted filename: Response to Reviewers.docx [file pone.0288089.s003.docx]

**Answer to the Reviewers’ reports on Manuscript ID PONE-D-22-35074R1 – “Mobile Phones and Infant Health at Birth.”**

Dear Associate Editor and Reviewers,

Thank you again for your deep engagement with our paper, which has now considerably improved as a result of the review process. In what follows, we have addressed the last two lingering concerns raised by Reviewer 2.

*Reviewer #2: The authors have addressed all of my concerns very well. The theoretical background and motivation for the mechanism analysis has been much improved and strengthened, and I appreciate the series of robustness checks that have been done. The new analyses in figures 4-6 much improve the paper.*

Thank you so much, we are really grateful for all the comments and suggestions.

*1. Page 7: “given that birth weight mostly reflects choices, behaviors, and constraints faced by mothers pre- and during pregnancy” While behavior is certainly an important determinant, I think that sentence is too strong. There are highly predictive maternal non-modifiable characteristics that affect birth weight including mother’s own birth weight, mother’s height, mother’s age, parity, etc.*

Thank you, we have revised and softened this sentence.

*2. The robustness check that includes mother’s weight as a control is important to highlight, given the large reduction in point estimate for birthweight and LBW. However, I do not see this robustness check referenced in the text or in the limitations as the authors stated. Please include this analysis in the appendix and add the text to the robustness checks paragraph and to the limitations discussion.*

Done, this robustness check has now been added to the paper and the lack of complete weight data discussed in the limitations section.
